# Pancreatic exocrine insufficiency

**DOI:** 10.1016/j.eclinm.2026.103880

**Published:** 2026-04-11

**Authors:** Daniel de la Iglesia, Miroslav Vujasinovic, J.-Matthias Löhr, J. Enrique Dominguez-Muñoz

**Affiliations:** a Department of Gastroenterology, University Hospital Alvaro Cunqueiro, Vigo, Galicia, Spain; b Galicia Sur Health Research Institute, Spain; c Department of Medicine, Karolinska Institutet, Sweden; d Department of Upper Abdominal Diseases Karolinska University Hospital, Stockholm, Sweden; e Department of Clinical Science, Karolinska Institutet, Sweden; f Department of Gastroenterology and Hepatology, University Hospital of Santiago de Compostela, Spain; g Research Health Institute of Santiago de Compostela (IDIS), Spain

**Keywords:** Pancreatic exocrine insufficiency, Pancreas, Chronic pancreatitis, Pancreatic cancer, Malabsorption, Pancreatic enzyme replacement therapy

## Abstract

Pancreatic exocrine insufficiency (PEI) is a common but frequently under-recognised condition that often presents with non-specific gastrointestinal symptoms, which may be subtle in early stages. Evidence from studies published between 2000 and 2026 indicates that, although the leading causes of PEI are pancreatic diseases such as chronic or autoimmune pancreatitis, pancreatic cancer, and pancreatic surgery, it is also increasingly recognised in common conditions including diabetes mellitus and ageing. Clinically, PEI should be suspected in patients presenting with diarrhoea, steatorrhoea, bloating, weight loss, or abdominal discomfort, particularly when malnutrition or micronutrient deficiencies are present. These deficiencies are associated with clinically relevant complications, including osteoporosis and cardiovascular disease, highlighting the importance of timely diagnosis and appropriate management. Pancreatic enzyme replacement therapy remains the cornerstone of treatment, with currently available porcine-derived formulations demonstrating high efficacy, although future research should explore alternative enzyme sources and novel therapeutic approaches.

**Funding:**

The authors received no financial support for the research, authorship, and/or publication of this article.

## Introduction

Pancreatic exocrine insufficiency (PEI) is a life-threatening condition. Regardless of the aetiology, patients with PEI are dependent on oral pancreatic enzyme preparations, similar to patients with endocrine pancreatic insufficiency dependent on insulin. As PEI is usually not reversible, patients require lifelong pancreatic enzyme replacement therapy (PERT). It is therefore essential to identify and correctly diagnose patients with PEI beyond those with pancreatic disease. The recently published evidence-based European guidelines for the diagnosis and treatment of PEI^1^ serve as a basis for this review.

## Search strategy and selection criteria

We searched MEDLINE (PubMed) and Embase from January 1, 2000 to January 2026 using combinations of the following terms: “pancreatic exocrine insufficiency”, “pancreatic enzyme replacement therapy”, “pancreatectomy”, “pancreaticoduodenectomy”, “pancreatic cancer”, “chronic pancreatitis”, “acute pancreatitis”, “diabetes”, “cystic fibrosis”, “fecal elastase”, and “13C breath test”. We prioritised systematic reviews, meta-analyses, randomised controlled trials, and large prospective cohort studies. Reference lists of relevant studies and recent international guidelines were manually screened to identify additional eligible publications.

## History

After the discovery of the pancreas as an organ, probably by Herophilos (300 BC), it took almost a thousand years to discover the pancreatic duct (Wirsung, 1643), the pancreatic juice (Regnier de Graaf, 1664) and its digestive capacity (Claude Bernard, 1856).^2^ Then, in 1859, J. Rutgers successfully treated the first patient with PEI using a crude extract of ground calf pancreas.^3^ Over the last 160 years, the manufacture and formulation of pancreatic enzyme preparations (pancreatin) has matured.^4^ Consensus on the use of pancreatin in chronic pancreatitis (CP) has evolved into evidence-based guidelines.^5^

## Definition and pathophysiology

With the recent European multidisciplinary guidelines, the definition of PEI changed towards a deficit in the function of pancreatic enzymes: while PEI still can be caused by a reduction in the exocrine pancreatic secretion, it also stipulates that the intraluminal activity of pancreatic enzymes below the level that allows normal digestion of nutrients is considered a cause of PEI.^1^ Both can cause maldigestion of nutrients that can lead to intestinal symptoms and/or nutritional deficiencies^1^

Three main factors explain the development of PEI in most patients. Failure of the exocrine pancreas to provide the necessary enzymes to the intestinal lumen for normal nutrient digestion is the main factor.^6^ Decreased pancreatic secretion may be secondary to loss of pancreatic parenchyma, as occurs in patients with CP, cystic fibrosis (CF), acute necrotising pancreatitis, or after surgical pancreatectomy, or to obstruction of the main pancreatic duct, which often occurs in patients with pancreatic cancer (PC).^7^ Reduced postprandial vagal (autonomic nerve division) and hormonal (low cholecystokinin [CCK] and secretin release) stimulation of pancreatic secretion, together with inappropriate mixing of chyme with digestive enzymes, are additional factors leading to PEI in patients following pancreaticoduodenectomy, gastrectomy, or gastric bypass.^8^

Conceptually, PEI should be regarded as a syndrome of clinically relevant maldigestion rather than as an isolated biochemical abnormality. Indirect tests such as fecal elastase-1 (FE-1) quantify pancreatic enzyme output but do not directly measure digestive capacity, and their correlation with fat absorption is variable. Reduced FE-1 values may therefore reflect decreased secretion without clinically meaningful malabsorption. Although staging models describing “exocrine pancreatic dysfunction” have been proposed to characterise early reductions in secretion,^9^ these stages may occur in individuals who remain clinically pancreas-sufficient and who do not require PERT. Accordingly, the diagnosis of PEI should be anchored to evidence of maldigestion—manifested by compatible symptoms and/or objective nutritional compromise—rather than based solely on secretion-based test abnormalities.

## Epidemiology

Studies of the prevalence of PEI in various diseases and conditions are biased by the pancreatic function test used. In these studies, PEI is often defined as reduced pancreatic secretion (e.g., as assessed by the FE-1), but secretion tests tend to overestimate the prevalence of PEI.^10^

**Fig. 1:**
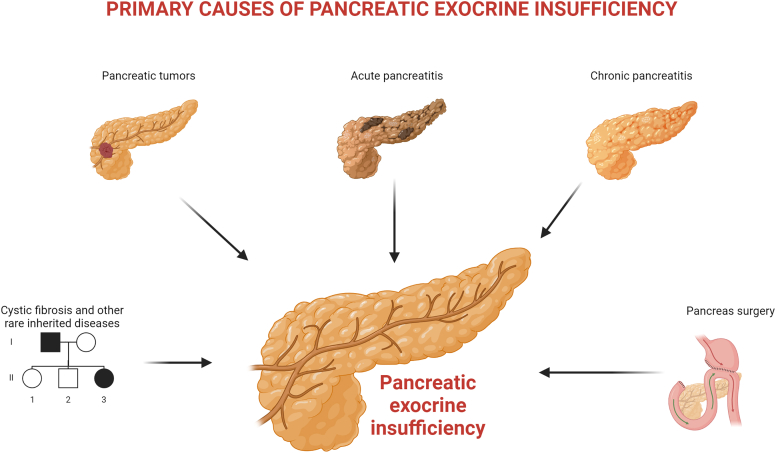
Primary causes of pancreatic exocrine insufficiency.

PEI is a common complication of pancreatic disease and surgery (Fig. 1). The prevalence of PEI in CP ranges from 20% to 90%, depending on the duration, severity, and aetiology of the disease,^11^ and more than 80% of patients with chronic calcific pancreatitis have PEI.^12^ PEI is also common after acute pancreatitis (AP), and 27–35% of patients have persistent PEI at long-term follow-up, especially those with alcoholic aetiology and necrotising and severe forms of the disease.^13^ Three out of four patients with advanced PC have PEI, and the risk of PEI in this population is three times higher if the tumour is located in the head of the pancreas compared to the body and tail.^14^ Most patients with CF and severe biallelic CFTR mutations develop PEI early in life, while the remaining patients develop PEI over time, mainly in association with the development of pancreatitis.^15^ The prevalence of PEI after pancreatic surgery (PS) varies mainly according to the surgical procedure and the condition of the pancreas before surgery. The development of PEI is the rule after total pancreatectomy and pancreaticoduodenectomy, whereas it varies according to the size of the pancreatic remnant after distal pancreatectomy.^16^ Subtotal gastrectomy results in altered fat digestion and absorption in about two thirds of patients, especially after Roux-en-Y reconstruction.^17^ The prevalence of PEI in other conditions, such as diabetes mellitus (DM), fatty pancreas and ageing pancreas, according to the new definition is unknown.^1^

## Diagnosis

Evaluation for PEI should be considered in individuals with established pancreatic disease (e.g., CP, PC, or following pancreatic resection) in those with other conditions predisposing to PEI (e.g., gastrectomy) and in patients presenting with otherwise unexplained features of maldigestion or nutritional compromise.^1^

### Clinical features and nutritional assessment

Symptoms such as steatorrhoea, increased stool volume, foul-smelling stools, bloating, flatulence, abdominal discomfort, and weight loss suggest PEI, but are neither sensitive nor specific. Notably, symptoms may be absent in patients who adapt their diet by reducing fat intake,^18^ and they often overlap with other gastrointestinal (GI) disorders, including celiac disease (CeD), irritable bowel syndrome (IBS), and small intestinal bacterial overgrowth.

Nutritional assessment is therefore an essential component in the evaluation, although detected abnormalities are also non-specific. Clinically relevant markers include anthropometric measures (body weight, body mass index, unintentional weight loss, mid-arm circumference and grip strength) and biochemical markers (albumin, haemoglobin, lymphocyte count, plasma proteins, fat-soluble vitamins and trace elements). These measures reflect disease burden and help guide enzyme supplementation, but they should not be used in isolation to diagnose PEI.^1^

In addition to clinical history and nutritional assessment, validated symptom-based instruments may assist in quantifying disease burden. The Pancreatic Exocrine Insufficiency Questionnaire (PEI-Q) is a validated patient-reported outcome tool designed to capture symptoms and quality-of-life impairment related to PEI.^19^ While it does not replace objective testing, the PEI-Q may be useful for baseline assessment, monitoring response to PERT, and facilitating structured follow-up in both clinical practice and research settings.

### Diagnostic methods

Different diagnostic methods for PEI are summarised in Table 1.

**Table 1:** Diagnostic tests for pancreatic exocrine insufficiency: strengths, limitations, and clinical use.

Test	What it measures	Strengths	Limitations/Pitfalls	Availability and clinical use
Coefficient of fat absorption (CFA)	Quantitative assessment of fat digestion and absorption during a 72-h stool collection on a 100 g fat diet	Considered the gold standard in clinical trials; regulatory reference for PERT efficacy	Laborious; poor patient acceptability; prone to dietary non-adherence; high laboratory burden; unsuitable for routine clinical use	Restricted to research and regulatory registration studies
^13^C-mixed triglyceride (^13^C-MTG) breath test	Lipase activity reflected by fat hydrolysis and subsequent^13^CO_2_ exhalation	High sensitivity and specificity (>90% vs CFA); non-invasive; well-tolerated by patients	Requires 4–6 h of breath sampling; results affected by pulmonary or hepatic dysfunction; limited availability	Recommended in centres where available; not yet widely implemented
Fecal elastase-1 (FE-1)	Concentration of pancreatic elastase in stool (μg/g)	Widely available; non-invasive; single stool sample; unaffected by PERT	Concentration-based—false-low results in watery stools; limited specificity at 100–200 μg/g; does not directly assess digestive capacity	First-line test in clinical practice; repeat if watery stool; interpret according to pre-test probability
Direct pancreatic function tests (secretin/CCK stimulation, endoscopic methods)	Bicarbonate and/or enzyme output following hormonal stimulation	Physiological assessment; considered research standard	Invasive, time-consuming, and expensive; limited availability; measures ductal rather than acinar function	Reserved for research settings; not recommended for routine PEI diagnosis
Serum nutritional markers (fat-soluble vitamins, albumin, micronutrients)	Downstream nutritional consequences of PEI	Useful for assessing deficiency burden and guiding supplementation	Low specificity; deficiencies may be multifactorial; delayed indicators of PEI	Employed as adjuncts in diagnosis and follow-up
Therapeutic trial of PERT	Clinical response to enzyme replacement therapy	Pragmatic, patient-centred approach	Non-specific clinical response; possible placebo effect; risk of overtreatment	Appropriate when diagnostic uncertainty persists, particularly in resource-limited settings

#### Digestion tests (CFA and ^13^C-MTG breath test)

According to the current definition, pancreatic function for the diagnosis of PEI is best assessed by digestion tests, including quantification of the coefficient of fat absorption (CFA) and the ^13^C-mixed triglyceride (MTG) breath test, when performed in the appropriate clinical context.

The CFA remains the standard requirement by US and European regulatory authorities to assess the efficacy of PERT in clinical trials.^20^ However, CFA has important limitations that restrict its clinical applicability. Patients must adhere a diet containing 100 g of fat per day for five days, and all stool must be collected over the final three days of this period. Both dietary adherence and stool collection are challenging for patients and handling of fecal samples in the laboratory is cumbersome and unpleasant. They have to collect all the faeces for the last three days of this five-day period, which, together with the handling of faeces in the laboratory, is very uncomfortable.

The ^13^C-MTG breath test was developed as a more convenient way to assess fat digestion for the diagnosis of PEI and to evaluate the efficacy of PERT in these patients.^6^ Compared with the CFA as the reference method, the ^13^C-MTG breath test has a sensitivity and specificity of more than 90% for the diagnosis of PEI.^21^ Although easily applicable in clinical practice, its availability remains limited in many countries.

#### Pancreatic secretion tests (direct invasive tests and faecal elastase test)

Due to the limited applicability and availability of digestion tests in clinical practice, pancreatic secretion tests are widely used to evaluate exocrine pancreatic function in PEI. Direct invasive tests, including the secretin-CCK test, the Lundh test and the endoscopic-based secretin-stimulated test, are not recommended for the diagnosis of PEI due to their invasiveness and high cost.^1^

FE-1 is the most frequently used pancreatic function test in clinical practice, as it is widely available and requires only a small stool sample for analysis.^22^ However, several important limitations affect its reliability. Because it is concentration-based assay, FE-1 levels are strongly influenced by stool consistency; in watery or loose stools, values are frequently spuriously low, potentially leading to false positive diagnoses of PEI. In addition, FE-1 does not measure digestive capacity but rather reflects pancreatic enzyme secretion, and values in the intermediate range (100–200 μg/g) lack specificity.^10^ Therefore, a low FE-1 result should always be interpreted in the context of the pre-test probability, nutritional status, and other relevant clinical findings.

#### Pragmatic clinical pathway

**Fig. 2:**
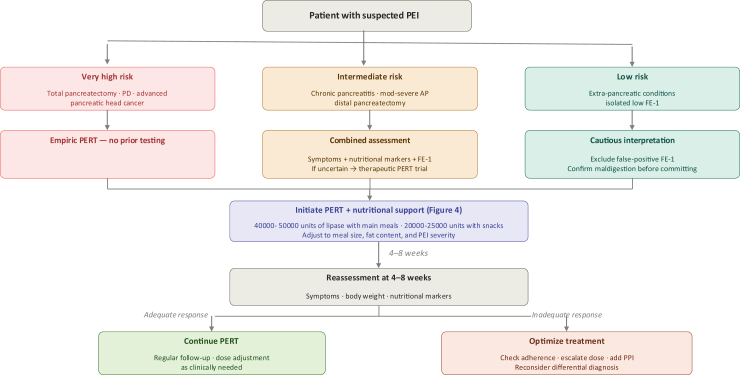
Risk-stratified diagnostic and treatment pathway for PEI. AP = acute pancreatitis; CP = chronic pancreatitis; FE-1 = faecal elastase-1; PD = pancreaticoduodenectomy; PERT = pancreatic enzyme replacement therapy; PPI = proton pump inhibitor.

For bedside applicability, a risk-stratified diagnostic and treatment approach is recommended and summarised in Fig. 2.

## Clinical symptoms and natural course of PEI

PEI is characterised by a wide range of clinical symptoms primarily due to maldigestion of nutrients, particularly fats, proteins and fat-soluble vitamins. The presentation and severity of symptoms can vary depending on the underlying aetiology, the degree of enzyme deficiency and the patient's dietary habits.

Steatorrhoea, presenting as bulky, pale, oily stools, is the most specific manifestation but typically occurs late, when lipase output falls below 10% of normal.^23^ Foul-smelling stools may be a more specific symptom, albeit rare.^24^ Many patients first experience non-specific symptoms such as abdominal pain, bloating, flatulence, and altered bowel habits,^18^ which overlap with other GI disorders and complicate diagnosis.

Malnutrition is a major concern in PEI and often precedes the onset of steatorrhoea.^25^ Patients may experience unintentional weight loss, muscle wasting and fatigue.^26^ Micronutrient deficiencies, particularly of the fat-soluble vitamins (A, D, E and K), are common in PEI and can lead to various complications if left untreated.^27^ Unless treated, PEI leads to severe malnutrition, increased risk of osteoporosis, sarcopenia and cardiovascular events.^28^ In addition, malnutrition in PEI is associated with reduced quality of life, increased hospitalisation rates and higher mortality.^29^

PEI affects not only physical but also psychosocial functioning with many patients reporting fatigue, reduced work productivity and limitations in social activities.^30^ This impairment often improves with appropriate treatment.^31^

In most conditions, PEI is progressive and irreversible, although partial recovery of pancreatic function may occur after AP. The development and progression of PEI is influenced by factors such as the underlying disease.^32^ Understanding these disease-specific patterns is crucial for appropriate surveillance and timely intervention.

Recent research has shed light on the potential role of the gut microbiome in PEI.^33^ Alterations in the gut microbiota have been observed in patients with PEI, which may contribute to maldigestion and GI symptoms.^34^ Reduced pancreatic enzyme secretion may alter the luminal environment, affecting the composition and function of the gut microbiome. Conversely, alterations in the microbiome may influence nutrient absorption and metabolism, suggesting that modulation of the gut microbiome may be a potential therapeutic target in the management of PEI.^35^

### PEI in chronic pancreatitis

CP is one of the most common causes of PEI (Table 2). The development of PEI in CP is primarily due to the progressive destruction of pancreatic acinar cells and obstruction of the pancreatic ducts, leading to reduced enzyme production and secretion.^36^ This process is typically gradual, with exocrine function declining over the years as the disease progresses.^37^ The rate of progression can vary significantly between individuals, influenced by factors such as aetiology, genetic predisposition and environmental factors.^38^

**Table 2:** Prevalence of pancreatic exocrine insufficiency across diseases and conditions.

Disease/Condition	Reported prevalence	Key determinants
Chronic pancreatitis	20–90%; cumulative risk 50% at 10 years, 80% at 20 years	Progressive acinar destruction, ductal obstruction, fibrosis; faster in alcoholic CP; genetic mutations (CFTR, SPINK1, PRSS1)
Acute pancreatitis	27–35%; up to 62% at index admission, improves over follow-up	Necrosis, fibrosis, severity, alcoholic aetiology, invasive interventions (e.g., necrosectomy)
Pancreatic cancer	50–90%, depending on tumour location and stage	Ductal obstruction, parenchymal loss, postsurgical changes
Cystic fibrosis	75–90%; up to 85% at birth in severe genotypes	CFTR mutations causing ductal obstruction and parenchymal loss
Autoimmune pancreatitis	∼45% at diagnosis; ∼36% during follow-up	Inflammation-mediated acinar destruction and ductal obstruction
Pancreatic surgery	80% after pancreaticoduodenectomy, ∼50% after distal pancreatectomy, 100% after total pancreatectomy	Loss of pancreatic mass, altered anatomy, reduced stimulation
Diabetes mellitus	10–50%; pooled prevalence in type 2 diabetes ∼22%	Reduced exocrine secretion
Coeliac disease	∼13.5% overall; 18% untreated vs 7% treated	Impaired CCK/secretin release
IBD	Reduced secretion in 41% by secretin-caerulein test; FE-1 usually normal	Mainly linked to AIP type 2
IBS	∼4.6% pooled	False positives due to diarrhoea
Drug-induced	SSA: steatorrhoea in ∼8%; ICIs: atrophy in 7.7%	Hormonal suppression; immune-mediated pancreatic damage
Inherited/genetic syndromes	Shwachman-Diamond: incidence 1:50,000; others include Johanson-Blizzard, Pearson, Shteyer, agenesis, HNF1B, CELA, CEL mutations	Congenital enzyme deficiency, acinar/ductal maldevelopment, gene defects
Fatty pancreas	9–56% with low FE-1	Pancreatic fat infiltration
Ageing	11.5% low FE-1 in population ≥50 years	Reduced enzyme/bicarbonate secretion
HIV infection	20–50%	Malnutrition, chronic infection, drug effects
Other systemic diseases	CKD, heart failure, Sjögren's: variable, low-quality evidence	Multifactorial, unclear mechanisms

CP: chronic pancreatitis; IBD: inflammatory bowel disease; IBS: irritable bowel syndrome; HIV: human immunodeficiency viruses; FE-1: fecal elastase test; SSA: somatostatin analogues; CKD: chronic kidney disease.

The alcoholic aetiology of CP is associated with a higher risk and more rapid progression of PEI than other aetiologies.^32^ The mechanisms underlying this increased risk may include direct toxic effects of alcohol on pancreatic acinar cells, increased oxidative stress and alterations in pancreatic stellate cell activation.^39^ Importantly, concomitant smoking further accelerates the course of alcohol-associated CP and contributes to the risk of developing PEI, highlighting the synergistic detrimental effects of these exposures. Mutations in the CFTR, SPINK1 and PRSS1 genes, among others, have been associated with an increased risk of CP.^40^ These genetic factors may influence the age of onset, rate of progression and severity of PEI, highlighting the importance of genetic testing in certain clinical scenarios.

In CP the presence of PEI has been independently linked to systemic complications such as osteoporosis, sarcopenia and cardiovascular events,^26^^,^^28^^,^^41^ as well as increased mortality.^29^

The relationship between PEI and pain in CP is bidirectional. PEI may contribute to pain in CP through altered gut–brain interactions and nutrient sensing.^42^ In addition, maldigestion associated with PEI may lead to changes in intestinal permeability, alterations in gut hormone secretion and activation of visceral nociceptors. Conversely, severe pain in CP may lead to reduced oral intake and exacerbate malnutrition associated with PEI.^43^ These findings highlight the complex interplay between PEI and other aspects of CP, and emphasise the need for comprehensive management strategies that address both pain and malnutrition.^30^

### PEI in other pancreatic diseases

Pancreatic diseases other than CP, mainly AP, PC, CF, autoimmune pancreatitis (AIP), fatty pancreas and PS, can also lead to PEI (Table 2).

PEI develops after AP in approximately one third of patients, depending on the severity of the initial attack and the method used to diagnose PEI. This prevalence is as high as 62% at the index admission for AP and decreases during follow-up.^13^ Traditionally, the development of PEI has been attributed to the extent of pancreatic necrosis and subsequent fibrosis, disease severity, alcoholic aetiology, and invasive treatments such as necrosectomy, which may impair the organ's ability to produce and secrete sufficient digestive enzymes.^13^ However, recent prospective studies have shown that clinically relevant PEI can also occur after mild AP, even in the absence of necrosis.^44^ These findings underscore the need for systematic assessment of exocrine function after every episode of AP. PEI in patients with AP may adversely affect functional recovery, prolong hospital stay and reduce quality of life.^45^

PEI in PC arises through several distinct mechanisms depending on disease stage and treatment.

In preoperative disease, obstruction of the main pancreatic duct—particularly in tumours located in the pancreatic head^46^—leads to upstream atrophy and impaired enzyme secretion. In post-resection patients, parenchymal loss and anatomical rearrangement further compound exocrine dysfunction.^47^ In advanced unresectable PC, tumour burden, cachexia, systemic inflammation, and biliary obstruction contribute to maldigestion and progressive nutritional decline.

Severe exocrine dysfunction, reflected by markedly reduced FE-1 levels, has been associated with poorer survival in observational studies.^48^ Although randomised controlled trials are lacking, multiple retrospective and prospective cohort studies suggest that PERT may improve nutritional status, stabilise weight, enhance tolerance to systemic therapy, and potentially influence survival.^14^ Beyond its effect on survival, the burden of PEI on quality of life in PC is increasingly recognised, with symptoms such as steatorrhoea, weight loss, and fatigue significantly reducing physical functioning and overall well-being.^49^ These data underscore the importance of early recognition and systematic management of PEI as part of comprehensive oncological care.^50^

PEI is a defining feature of CF, affecting up to 75–90% of patients.^15^ Exocrine insufficiency in CF results from impaired chloride transport due to CFTR mutations, leading to thick, viscous secretions that obstruct the pancreatic ducts with consequent loss of pancreatic parenchyma.^51^ Unlike other causes, PEI in CF often manifests early in life, with up to 85% of patients having PEI at birth.^52^ CF genotype correlates with the risk of PEI.^53^ Patients with two severe biallelic (class I, II, III and IV) CFTR mutations develop PEI early in life. Recent advances in CF management, including the introduction of CFTR modulators, have raised questions about their impact on pancreatic function. Pancreatic secretion improves during treatment with ivacaftor in young children with gating mutations,^54^ but the long-term effects of these therapies on PEI in CF remain to be elucidated. The management of PEI in CF requires a multidisciplinary strategy including PERT, nutritional support, and monitoring. PERT dosing should be individualised with regular evaluation of nutritional status.^1^

AIP is a distinct form of pancreatitis that can lead to PEI.^55^ The mechanisms of PEI in AIP are not fully understood but are likely to involve inflammation-mediated acinar cell destruction and ductal obstruction. The evolution of PEI in AIP may differ from that in other pancreatic disorders, since it can be reversible with adequate corticosteroid treatmen.^56^ This highlights the importance of early diagnosis and treatment of AIP to prevent irreversible pancreatic damage.

The relationship between fatty pancreas and PEI remains uncertain.^57^ MRI studies suggest an inverse association between pancreatic fat content and FE-1 levels.^57^ However, patients with fatty pancreas and low FE-1 levels are usually asymptomatic and have normal ^13^C-MTG-breath test results and normal serum nutritional panel.^57^ Therefore, the presence of fatty pancreas should be interpreted within the broader clinical and metabolic context rather than considered an independent determinant of maldigestive dysfunction.^57^

PEI is a frequent and often under-recognised consequence of PS, with prevalence and severity strongly influenced by the type of resection, baseline glandular function, and postoperative anatomy.^58^

After total pancreatectomy, clinically relevant PEI is universal and permanent, and diagnostic confirmation is unnecessary.

Following pancreaticoduodenectomy (PD), biochemical evidence of exocrine dysfunction is highly prevalent, and most patients develop clinically relevant PEI, although severity varies according to remnant volume, preoperative pancreatic texture, duct diameter, and adjuvant therapy. Mechanisms include parenchymal loss, disruption of duodenal hormone-mediated stimulation (CCK and secretin), postcibal asynchrony, and altered gastric emptying.^59^ Importantly, PEI may evolve over time after PD, reflecting progressive fibrosis and functional decline in the remnant pancreas, underscoring the need for longitudinal reassessment rather than single time-point testing.

After distal pancreatectomy, the risk of PEI correlates with remnant pancreatic volume and pre-existing pancreatic disease. Patients operated for PC or CP have a higher risk than those undergoing resection for benign lesions.

A critical limitation of the surgical literature is that many studies define PEI based on symptoms alone, while others rely exclusively on FE-1 measurements without confirming maldigestion.^58^ Because postoperative gastrointestinal symptoms are common and non-specific and secretion-based tests do not directly measure digestive capacity, these approaches may not accurately reflect clinically meaningful maldigestion. Consequently, reported prevalence estimates of PEI vary substantially across studies.

Despite the high prevalence of exocrine dysfunction following pancreatic resection, real-world data consistently demonstrate substantial undertreatment: in a large US population-based study of patients with pancreatic cancer only 21.9% filled a PERT prescription and only 5.5% received an adequate dose,^60^ highlighting the profound gap between guideline recommendations and clinical practice.

From a survivorship perspective, untreated PEI may impair nutritional recovery, contribute to sarcopenia, reduce tolerance to adjuvant chemotherapy, and negatively affect quality of life.^1^ Therefore, in high-risk surgical populations—particularly after total pancreatectomy or PD—empiric initiation of PERT with structured clinical reassessment is appropriate, even in the absence of formal digestion testing.

### PEI in other conditions

**Fig. 3:**
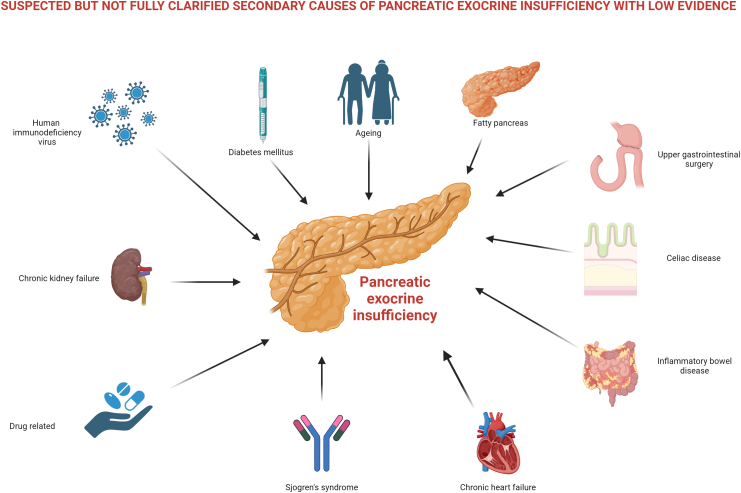
Causes of secondary pancreatic exocrine insufficiency.

In addition to pancreatic diseases, several extra-pancreatic conditions have been linked to PEI, although evidence remains inconsistent and often based on indirect tests rather than demonstration of true maldigestion (Fig. 3). Reported prevalence estimates for these conditions are summarised in Table 2.

Afferent and efferent bowel loop syndromes may develop following upper GI surgery, which, in addition to accelerated intestinal transit time and colonisation of the upper GI tract by pathogenic bacteria, may result in inadequate stimulation and poorly synchronised pancreatic enzyme secretion, a condition known as postcibal asynchrony.^61^ Postcibal asynchrony can cause PEI due to a combination of impaired postprandial stimulation of pancreatic secretion and asynchrony between gastric emptying of nutrients and biliopancreatic secretion.

Reduced exocrine secretion, reflected by low FE-1 levels, is observed more often in individuals with DM than in healthy controls. A meta-analysis estimated a pooled prevalence of 22% in type 2 DM,^62^ although lower rates (around 5%) have been reported when patients with concomitant CP were excluded.^63^ Despite this increased frequency, routine screening for PEI in DM is not recommended.^1^ Instead, evaluation should be directed towards patients with symptoms of maldigestion or when type 3c DM is suspected, where targeted assessment of exocrine function is warranted.

Age-related changes in pancreatic morphology and perfusion are well recognised, but their impact on exocrine function remains uncertain.^64^ The largest population-based study, including 914 individuals aged 50–75 years, reported low FE-1 levels in 11.5% of participants.^65^ The clinical relevance of these findings is unclear, but when PEI is diagnosed in elderly patients, it should be managed according to the same principles as in younger populations.

In CeD, PEI is thought to result from impaired release of cholecystokinin and secretin, while pancreatic morphology and intrinsic function remain largely intact. A recent meta-analysis reported a pooled prevalence of 13.5% in biopsy-proven CeD, with higher rates in untreated patients (18.2%) compared with those adhering to a gluten-free diet (6.9%).^66^ Routine pancreatic function testing is not recommended at diagnosis, but FE-1 assessment may be valuable in patients with persistent symptoms despite dietary adherence.^1^

Secretin-caerulein testing demonstrates reduced pancreatic secretion in 41% of inflammatory bowel disease (IBD) patients,^1^ while FE-1 levels remain normal in Crohn's disease patients. PEI in IBD appears primarily associated with AIP type 2, with low FE-1 levels reported in 19%^67^ and 31%^68^ of patients with concurrent IBD and AIP.

A pooled prevalence of 4.6% has been reported in IBS patients,^69^ but this likely represents an overestimation, as most studies did not include pancreatic imaging to exclude underlying pancreatic disease. False-positive FE-1 results should be considered in diarrhoea-predominant IBS patients without pancreatic pathology.

Drug-induced PEI has also been reported. Somatostatin analogues significantly reduce pancreatic enzyme secretion and inhibit regulatory hormone release.^70^ Immune checkpoint inhibitors cause pancreatic atrophy in 7.7% of cancer patients, with 1.1% developing PEI responsive to PERT.^71^

Shwachman-Bodian-Diamond syndrome is the second most common inherited PEI cause after CF (incidence 1:50,000). Other genetic conditions include Johanson-Blizzard, Pearson, and Shteyer syndromes, pancreatic agenesis, HNF1B mutations, isolated enzyme deficiencies, and CELA/CEL gene mutations causing combined diabetes and exocrine dysfunction.^1^

Chronic HIV infection is associated with symptomatic PEI in 20–50% of patients,^1^ while PEI prevalence in bacterial/parasitic infections remains poorly defined. PEI has also been reported with chronic kidney disease, chronic heart failure, and Sjögren's syndrome, though evidence quality is limited.^1^

A critical limitation of the existing literature is the inconsistent use of diagnostic criteria, with many studies relying solely on abnormal FE-1 results to define PEI.^1^ This approach fails to distinguish between subclinical pancreatic dysfunction and clinically relevant PEI, leading to potential misdiagnosis and biased outcomes. Given recent European guidelines emphasising more rigorous PEI definitions,^1^ the true prevalence across extra-pancreatic conditions remains largely unknown, highlighting the need for well-designed epidemiological studies applying current diagnostic recommendations.^1^

## Therapeutic approach to PEI

Regardless of the underlying condition mentioned above, once PEI is diagnosed, it should always be treated.^1^ The two main pillars of the therapeutic approach to PEI are nutritional advice and support and PERT. The general benefits of PERT in patients with PEI are numerous and include improving fat and protein absorption, which has a positive effect on body weight, nutritional status, symptoms and quality of life.^1^

Pancreatic enzyme preparations, particularly pancreatin, are the recommended first-line treatment for PEI.^1^ Commercially available pancreatic enzyme formulations are mainly in the form of pellets, tablets or powder. Smaller pellets are preferred because of their demonstrated clinical efficacy; their reduced particles size allows for better mixing with food in the stomach and promotes simultaneous emptying with chyme from the stomach into the duodenum.^5^ PERT is available in a variety of preparations with different levels of lipase, amylase and protease activity; they are labelled according to their lipase activity.

PERT preparations are typically protected from gastric acid by an enteric coating, which ensures the enzymes remain intact until they reach the duodenum, where the coating dissolves at a pH of 5.5 or higher, allowing enzyme release.^72^ Preparations that have demonstrated efficacy are derived from porcine sources. Patients should be made aware of the porcine origin of these preparations before starting therapy, particularly for considerations related to lifestyle or dietary preferences (e.g., vegan or vegetarian) or religious beliefs.^1^

The initial dose of PERT depends on the age of the patient (adult or child), the severity of PEI (clinical symptoms and malnutrition), and the calorie and fat content of the meal.

**Fig. 4:**
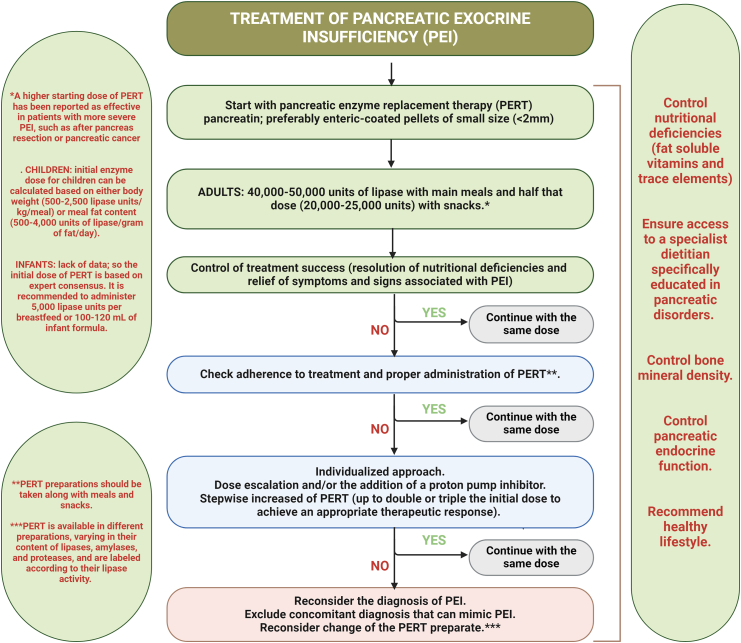
Treatment of pancreatic exocrine insufficiency (PEI). PERT = pancreatic enzyme replacement therapy.

The initial dose of lipase should correspond to at least 10% of the typical physiologic secretion.^5^ Research indicates that administering a minimum dose of 40,000–50,000 units of lipase with main meals and approximately half that amount (20,000–25,000 units) with snacks is effective in adult patients^1^ (Fig. 4). In individuals with more severe PEI, such as those who have undergone pancreatic resection or are dealing with PC, a higher starting dose of PERT may be beneficial.^1^ As a result, the PERT dosage should be individualised based on the severity of PEI as well as the patient's dietary patterns, including the quantity, caloric content, and fat composition of the meals.^1^ PERT preparations should be taken with meals and snacks to optimise its efficacy.^1^ In some cases, the addition of a proton pump inhibitor (PPI) may be necessary, particularly in patients with an inadequate response to PERT, as the acidic pH of the intraluminal gut significantly impacts the efficacy of PERT.^1^ Limited evidence exists regarding the practice of opening PERT capsules and ingesting the pellets with acidic liquids or semi-solid foods (pH < 5.5) in post-gastrectomy patients. This method may potentially enhance the mixing of the pellets with the food, though further research is needed to confirm its efficacy.^1^

The initial enzyme dosage in children can be determined based on either body weight (500–2500 lipase units/kg/meal) or the fat content of meals (500–4000 lipase units per gram of fat per day).^1^ In the absence of specific data, the initial dose of PERT in infants is typically guided by expert consensus. It is generally recommended to administer 5000 lipase units per breastfeeding session or per 100–120 ml of infant formula.^1^

Treatment response should be evaluated systematically, with success defined by resolution of PEI-related symptoms and correction of nutritional deficiencies. A formal clinical and nutritional reassessment is recommended at 4–8 weeks after PERT initiation across all risk groups, providing a structured opportunity to evaluate treatment adequacy, optimise dosing, and reconsider the diagnosis if response is absent or incomplete.^1^ In patients who do not respond adequately, adherence and correct administration should be confirmed before escalating therapy. Subsequent steps include dose escalation and, where not already prescribed, the addition of a proton pump inhibitor to improve the intraluminal pH environment for enzyme activity. In patients who remain symptomatic despite optimised PERT and acid suppression, alternative or coexisting conditions that may mimic or compound PEI—including small intestinal bacterial overgrowth, coeliac disease, or bile acid malabsorption—should be systematically excluded.

PERT is generally not associated with significant adverse effects. However, the use of high doses of older pancreatic enzyme products in patients with CF may increase the risk of fibrotic colonopathy. In children with CF, hyperuricosuria has been observed in a dose-dependent manner, attributed to the high purine content of the drug. Consequently, PERT should be administered with caution in patients with gout, hyperuricemia, or renal impairment. No drug–drug interactions have been reported and there is no evidence of adverse effects from PERT during pregnancy or lactation. Efficacy studies of PERT are primarily conducted in patients receiving oral nutrition. However, there is limited evidence available for patients with PEI who require enteral nutrition, where PERT must be delivered through a feeding tube.^1^ In this context, immobilised lipase cartridges have been developed to hydrolyse fats directly within enteral formulae before administration, bypassing the challenges of enzyme survival in the GI tract. Early studies in CF and short bowel syndrome suggest feasibility and improved fat absorption,^73^ though their role in routine clinical practice remains to be defined.

In patients with dysphagia, swallowing PERT capsules may be challenging. Although evidence on this matter is scarce, the consensus recommendation is to suspend PERT in acidic puréed foods when capsules cannot be ingested.

For patients with PEI on adequate PERT, dietary fat restriction is generally unnecessary.^1^ In cases of malnutrition, overly restrictive diets may be counterproductive. Therefore, patients with PEI should be encouraged to follow a varied, balanced diet while adhering to an appropriate PERT dosage. Additionally, patients with PEI should have access to a dietitian with specialised training in pancreatic disorders.^1^^,^^5^

## Different pancreatin preparations

The requirements for the “ideal” pancreatin were proposed as early as 1879: 1) be active on all three main food components (protein, fat, carbohydrate); 2) withstand gastric acid; 3) be acceptable to the patient.^74^ Enzymes have evolved from the ground gland to powder (the first commercial pancreatin) and tablets to the microparticles coated with an acid-resistant film and encapsulated in gelatine capsules (Fig. 4). Broadly speaking, these pancreatin preparations are comparable and represent the peak of evolution based on the natural source, but differences in particle size and stability of the enteric coating at acidic pH may affect efficacy.^1^ A recent development, not yet fully evaluated, is the addition of bicarbonate to create a more alkaline moiety near the pancreatin. Due to recent reports on exogenous factors influencing the safety and effectiveness of pancreatin, the search for non-porcine pancreatic enzyme preparations needs to be intensified.^75^ This is even more pressing since the source of the natural product had some periodic shortage.^76^

## Conclusion

PEI is a clinically important and frequently under-recognised condition that arises primarily in the context of pancreatic diseases and PS. Increasing evidence suggests that reduced pancreatic enzyme secretion may also occur in several extra-pancreatic conditions; however, the prevalence of clinically meaningful maldigestion in these settings remains uncertain and requires confirmation using digestion-based diagnostic tests. Accordingly, abnormal results from indirect secretion tests should not automatically be interpreted as evidence of clinically relevant PEI.

Distinguishing biochemical pancreatic dysfunction from clinically meaningful maldigestion is essential to avoid both underdiagnosis and inappropriate treatment. When left untreated, PEI contributes to progressive malnutrition, weight loss, sarcopenia, micronutrient deficiencies, osteoporosis, impaired quality of life, and—in oncologic populations—reduced tolerance to systemic therapies and potentially reduced survival.

Improved clinical awareness, systematic risk-based assessment, and timely initiation of PERT, integrated into structured and longitudinal care pathways, are therefore central to optimising nutritional status, quality of life, and overall outcomes in patients at risk of PEI.

## Outstanding questions

1. Can we identify novel, non-invasive biomarkers that correlate better with fat malabsorption than FE-1, particularly for mild-to-moderate PEI?
2. What is the true prevalence of clinically meaningful PEI in extra-pancreatic conditions when digestion-based tests are systematically applied?
3. Does PERT restore the gut microbiome composition in PEI patients, and does this restoration correlate with improved clinical outcomes?
4. What is the optimal enzyme dosing strategy across different clinical scenarios, including post-pancreaticoduodenectomy and advanced PC?
5. Can effective non-porcine enzyme formulations be developed to ensure sustainable global access to therapy?
6. Which patient-reported outcomes and nutritional markers best capture clinically meaningful treatment response?

Addressing these questions will refine patient selection, optimise treatment strategies, and improve the precision of PEI management.

## Contributors

All authors contribute equally in the design, writing and review of the manuscript. All authors accessed and verified the underlying data. All authors approved the final version of the manuscript and accept responsibility for the decision to submit the manuscript for publication.

## Data sharing statement

No new data were generated or analysed in this study. All information included in this review was derived from previously published studies cited in the manuscript. Therefore, no datasets are available for sharing.

## Declaration of interests

E.D.M reports honoraria for lectures or educational activities from Abbott and Viatris.

J.M.L. reports consulting fees from Falk Pharma; honoraria for lectures or educational activities from Amgen, Abbott, Viatris, Nordmark, and Frostpharma; stock ownership in Centogene and Pharmacyte; and serves as President of United European Gastroenterology (unpaid).

M.V. reports consulting fees from Abbott and Amgen and honoraria for lectures or educational activities from Abbott, Amgen, Nordmark Pharma, and Viatris.

D.I. reports honoraria for lectures or educational activities from Abbott and Viatris
